# A New Vision for *IJERPH*—A Transdisciplinary Journal of Environmental Research and Public Health

**DOI:** 10.3390/ijerph20186728

**Published:** 2023-09-07

**Authors:** Paul B. Tchounwou

**Affiliations:** RCMI Center for Urban Health Disparities Research and Innovation, Morgan State University, Baltimore, MD 21251, USA; paul.tchounwou@morgan.edu

Since its establishment in 2004, the *International Journal of Environmental Research and Public Health* (*IJERPH*) [1] has emerged as one of the top journals in the transdisciplinary field of environmental and public health sciences. For the past two decades, *IJERPH* has significantly contributed to scientific advances that have made and continue to make significant impacts toward improving environmental quality and human health. Up until 30 June 2023, over 60,000 papers had been published, including more than 17,000 cited at least 10 times and/or highlighted over 28,000 times in prominent news outlets such as *Forbes*, *The New York Times*, *The Washington Post*, *BBC News*, *National Geographic,* and *The Guardian.* Also, according to Google Scholar’s latest metrics released in July 2023, *IJERPH* ranks No. 1 in the Public Health category.

Recognizing the broad scope and multifaced nature of environmental research and public health, as well as the importance of ensuring that *IJERPH* continues to attract and publish the best research and novel discoveries in this field of scientific endeavor, a special editorial board meeting was held in Basel, Switzerland, on 5 July 2023. Dr. Paul Tchounwou, the founding Editor-in-Chief of *IJERPH*, along with Dr. William Douglas Evans (Section Editor-in-Chief—SEiC for the Section “Health Communication and Informatics”), Dr. Jimmy T. Efird (SEiC for the Section “Public Health Statistics and Risk Assessment”), Dr. Karl Goodkin (SEiC for the Section “Mental Health”), Dr. German Vicente Rodriguez (SEiC for the Section “Adolescents”), Dr. William Toscano (SEiC for the Section “Environmental Health”), several MDPI personnel including Dr. Shu-Kun Lin (President), Dr. Yugo Lin (COO), Stefan Tochev (CEO), Peter Roth (Head of Publishing), the Scientific Officers, the journal’s editorial team, and several others attended the meeting (Figure 1).

The overarching goals of this board meeting were to: (1) review the status of the journal; (2) identify strengths and weaknesses; and (3) brainstorm and make recommendations on strategic activities aimed at refining the scope and specific aims, improving the scientific quality, and enhancing the reputation of our journal.

Overall, this editorial board meeting was successful and highly productive. Great and thoughtful ideas were generated about the best strategies to address the decision from Clarivate to remove *IJERPH* from the list of impact factor journals based on the speculation that a couple of published papers did not fit the scope of *IJERPH* [2]. Also, several recommendations were made on key actions to be taken to continue to improve the scientific strength and societal impact of our journal.

## 1. New *IJERPH* Scope and Aims

The new scope and specific aims of *IJERPH* [3] stated below have been refined to focus more on its transdisciplinary nature, given the limitations of specific scientific disciplines toward conducting environmental and public health research in a comprehensive manner.

*IJERPH* is a peer-reviewed, transdisciplinary journal focused on publishing content related to health promotion and disease prevention. *IJERPH* aims to bring together all scientific communities from various disciplines that address health promotion, wellbeing, and the improvement of quality of life. We strive to broaden the perception of public health to encompass all aspects of biological, social, environmental, and behavioral determinants of health, shifting the focus beyond traditional boundaries.

The concept of environmental research within *IJERPH* should not be limited to an ecological perspective alone. Instead, it should be understood in a broader context, encompassing the interplay between human beings and their physical, mental, and social environments and their impact on health.

All studies submitted to *IJERPH* are expected to highlight how the results have a direct impact on health promotion. Authors are encouraged to publish their experimental and theoretical results in as much detail as possible. The full experimental details must be provided so that the results can be reproduced. *IJERPH* welcomes original articles, critical reviews, and short communications.

Although research on health care systems and their influence on public health is welcome, all regional studies should be framed within a global context. Please note that clinical studies not sufficiently addressing the aim of the journal will not be considered.

By adopting a transdisciplinary approach, *IJERPH* seeks to foster collaboration and knowledge exchange among diverse fields, contributing to a holistic understanding of health promotion and disease prevention.

## 2. New *IJERPH* Sections

Based on the revised scope and specific aims stated above, the editorial board made a recommendation to consolidate the sections of the journal by restructuring them into seven main sections. It was also recommended to appoint each of the current SEiCs who will not have an individual section to serve as a Co-SEiC. Serving in this capacity will help to ensure that the quality control and assurance of his/her sub-section are maintained.

The list of the new consolidated sections is as follows: (1) Global Health; (2) Healthcare Sciences; (3) Behavioral and Mental Health; (4) Infectious Diseases, Chronic Diseases, and Disease Prevention; (5) Exercise and Health-Related Quality of Life; (6) Environmental Health; and (7) Environmental Sciences. The revision and launch of the new sections will be completed in due course.

As the Editor-in-Chief, I fully recognize the value our authors place in getting their research published in a high-impact and well-respected journal. I will continue to work tirelessly with our editorial board to move our journal back to Clarivate in the next two years. In doing so, we will continue to maintain a rigorous and efficient peer-review process while paying close attention to all submissions to ensure that all manuscripts are carefully pre-checked for relevance and appropriateness prior to processing for a full peer-review. Also, we will screen all future proposals for Special Issues [4] or Topics [5] to ensure that they fit within the new scope of *IJERPH*.

I would like to acknowledge the outstanding contributions of the entire editorial board and the MDPI leadership team. I sincerely thank them for their valuable input in evaluating the Journal’s current status and recommending key strategies for moving *IJERPH* to the next level of scientific excellence. Their thoughtful advice and input on the strategic development of our journal are highly appreciated.

## Figures and Tables

**Figure 1:**
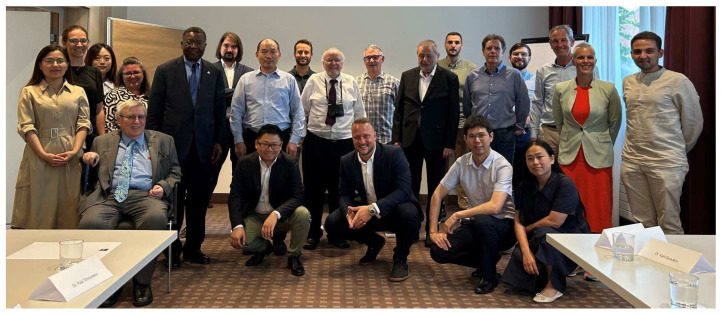
The attendees of the editorial board meeting on 5 July 2023.

## References

[1] *International Journal of Environmental Research and Public Health (IJERPH)* Home Page. https://www.mdpi.com/journal/ijerph.

[2] Clarivate Discontinues *IJERPH* and *JRFM* Coverage in Web of Science (Update). https://www.mdpi.com/journal/ijerph/announcements/5628.

[3] About *IJERPH*, Aims and Scope. https://www.mdpi.com/journal/ijerph/about.

[4] *IJERPH* Special Issues. https://www.mdpi.com/journal/ijerph/special_issues.

[5] MDPI Topics. https://www.mdpi.com/topics.

